# A Case of Post-Partum Hemorrhage with Unusual Complications—Recovery

**Published:** 1878-11-20

**Authors:** S. H. Anderson

**Affiliations:** Missouri


### SOUTHERN MEDICAL RECORD.

VOL. VIII. ATLANTA, GA., NOV. 20, 1878. NUMBER 11.

EDITORS:

THOMAS S. POWELL, M. D. W. T. GOLDSMITH, M. D. R. C. WORD, M. D.

*R. C. WORD, M. D., Managing Editor.*

SUBSCRIPTION—$2.00 PER ANNUM, IN ADVANCE.

☞ All Communications and Letters on Business connected with THE RECORD, for 1877 and 1878, must be addressed to the Managing Editor.

### *ORIGINAL AND SELECTED.*

### A CASE OF POST-PARTUM HEMORRHAGE WITH UNUSUAL COMPLICATIONS—RECOVERY.

BY. S. H. ANDERSON, M. D., OF MO.

Called on Mrs. D. July, 1878; second confinement; first labor having been normal in every respect; was told that Mrs. D. had, that morning, done a heavy washing for the family; arrived at full term of pregnancy; found her lying on her bed very restless, and unable to remain in one position but for a few minutes at a time. On making digital examination, found head presenting, but something very unusual in *shape* and *feel*, and after a thorough and careful examination of presenting part, I was satisfied that a large portion of the skull was wanting, as I could put my fingers down into a cup-like cavity, lined, as it were with the scalp. Having stated to the friends of the patient that something very different from the usual forms of labor might be expected, and having administered a full dose of morphia, I placed myself at the bedside of my patient, watching the progress of the labor with much interest. The membranes ruptured; the labor in second stage, and little progress being made, I sought for the cause of delay, which I found to be this: The rim of the cup-like cavity had caught on the *ossa pubis* and thus could make no further progress. The remedy was obvious, and fortunately, of easy accomplishment, which was merely to lift the cranium, and during a pain, prevent it catching, thus aiding the head to pass on and become engaged in the pelvic strait. Thus, after freeing the cranium from the *ossa pubis*, it readily passed, being collapsed to nearly half the size of a normal head. Not so, however, the body of the fœtus, the abdomen being enormously distended with gas, the result of decomposition. At first I thought of puncturing the abdomen in order to give exit to this gas ; but, on reflection, I resorted to *traction*, thus accomplishing delivery without any great amount of force. Placenta found loose in vagina and removed without anything very remarkable save that it and fœtus were both putrid. On examing child I found nearly all of the bones, constituting upper portion of the cranium wanting —scalp entire. The child had evidently been dead for some time—a week or more, perhaps, as the cutis readily separated. The child was small, and lower extremities deformed--*talipes-varus*.

Having remained with my patient a few hours, I mounted my horse and started for home, a distance of five miles or more. But I had not proceeded more than a few hundred yards from the house, when I was called back by the screams of the family. Starting my horse on a gallop, I soon reached the house. On entering, the first thing that attracted my attention was a loud gurgling sound resembling the noise made by pouring water or other liquid out of a keg or jug when quite full. The next thing attracting my attention, was quite a rivulet of blood running from the bed to the end of the house, ten or twelve feet away. Fortunately for my patient and myself, a bucket of coal water was at hand. Seizing a table-cloth folded up and lying on the table, I immersed it in the water and applied it to my patients abdomen without any wringing. Next, I called to the affrighted parents, who had incontinently fled from the house to assist me in raising foot of bedstead under which I placed blocks of wood in in order to elevate pelvis. And next, I sought for my ergot, when, lo! I had none with me. Doing the next best thing, I gave my patient a large dose of sugar lead and opium. By these means I succeeded in checking the most fearful *post-partum* hemorrhage it was ever my lot to witnesss. Also I had grasped the abdomen and womb in my hands and kneading them, somewhat like a woman, working dough, which materially, I think, assisted contraction. As before remarked, I succeeded in checking hemorrhage with remedies as indicated, but did not get perfect control of the flooding until I had sent for and received a supply of ergot from home. At several times during the night, I thought that my patient would die. She was totally blind for several hours and pulseless, cold and pale as the dead! For twelve hours I stood on my feet and fought death for a young and noble life, and had for my reward the intense gratification of seeing slowly, gradually, the hues of life returning to her corpse-like face, and light to her glazed eye.

No one but the true physician can know and feel the emotions of joy and self-gratulation afforded by the knowledge that himself, under God has been the means of snatching lovely woman in her hour of peril from the grasp of the fell destroyer.

In conclusion some may doubt my veracity as to the gurgling sound made by the blood pouring from the wound, but I solemnly experienced its truth, and which I can verify by the parents of the patient, who are worthy christian folks.

